# Challenging Retrieval of a Migrated Peripheral Venous Cannula Fragment in an Obstetric Patient: Case Report

**DOI:** 10.3390/life16050717

**Published:** 2026-04-23

**Authors:** Janos Szederjesi, Leonard Azamfirei, János Levente Turos, Emil Marian Arbănași, Gabriela Șalari, Matild Keresztes

**Affiliations:** 1 Department of Anesthesiology and Intensive Care, George Emil Palade University of Medicine, Pharmacy, Science and Technology of Târgu-Mureș, 540139 Târgu Mureș, Romania; yangzi37@gmail.com (J.S.); leonard.azamfirei@umfst.ro (L.A.); 2 1st Department of Obstetrics and Gynecology, George Emil Palade University of Medicine, Pharmacy, Science and Technology of Târgu-Mureș, 540139 Târgu Mureș, Romania; janos.turos@umfst.ro; 3 Vascular Surgery Department, George Emil Palade University of Medicine, Pharmacy, Science and Technology of Târgu-Mureș, 540139 Târgu Mureș, Romania; emil.arbanasi@umfst.ro; 4 Doctoral School of Medicine and Pharmacy, George Emil Palade University of Medicine, Pharmacy, Science and Technology of Târgu Mureș, 540139 Târgu Mureș, Romania; keresztes.matild.25@stud.umfst.ro

**Keywords:** catheter fracture, obstetric, catheter migration, cephalic vein

## Abstract

Intravenous cannulation is a routine procedure in clinical practice but may rarely be complicated by catheter fracture with intravascular fragment retention. Management is particularly challenging in obstetric patients, where both maternal safety and procedural risks must be carefully balanced. We report the case of a 31-year-old pregnant woman at 21 weeks of gestation admitted for conservative management of preterm prelabor rupture of membranes. Three days after peripheral intravenous catheter placement in the right cephalic vein, catheter fracture with intravascular retention of a fragment was identified. Ultrasound localized the fragment within the cephalic vein, with subsequent migration to the deltopectoral groove. An initial surgical exploration was unsuccessful. Following repeat ultrasound localization, a second surgical procedure performed by an experienced vascular surgeon enabled successful retrieval using a Fogarty catheter. Cephalic vein ligation was performed to prevent further migration. The procedure was completed without complications, and the pregnancy progressed for an additional nine weeks, culminating in preterm delivery of a viable neonate. This case highlights the importance of accurate localization, the need for a stepwise surgical approach after failed initial intervention, and the role of vascular expertise in achieving successful outcomes in complex obstetric patients.

## 1. Introduction

Intravenous cannulation is one of the most frequently performed invasive procedures in clinical practice, providing critical access for fluid administration, medication delivery, blood sampling, and emergency therapies [1,2]. Peripheral intravenous cannulas are favored for their ease of insertion, lower cost, relative safety, and ubiquity in wards, emergency departments, and perioperative settings. However, despite being viewed as a “routine” procedure, peripheral cannulas are not without risk [3,4,5].

One of the potentially serious complications is cannula fracture with retention of a fragment inside the vascular system, leading to an intravascular foreign body. These complications are uncommon but differ by device type, occurring with central venous catheters, peripherally inserted central lines, and port-a-caths, whereas peripheral intravenous catheter fractures are exceedingly rare and predominantly described only in isolated case reports [6]. The presence of such a fragment may result in migration to more central veins, the heart, or pulmonary arteries, and may cause arrhythmias, thrombosis, infection, or even vascular injury [3,7,8,9,10].

In the case of a pregnant patient, the situation is further complicated. Peripheral intravenous cannulation can be challenging in pregnancy due to edema that obscures venous landmarks, vasodilation with increased blood volume that alters vein tone, and gestational weight gain that increases subcutaneous tissue thickness [2,11,12].

Diagnosis of a fractured peripheral intravenous catheter is usually suspected when the catheter tip is missing after removal or when symptoms such as pain, swelling, or catheter dysfunction occur. Imaging modality selection depends on the radiographic properties of the catheter. Peripheral intravenous catheters are often radiolucent, in which case ultrasound is the preferred modality for localization, while plain radiography may be inconclusive. However, if the catheter has radiopaque components, radiography may also provide useful information regarding its location.

Management of a fractured peripheral intravenous catheter depends on the location of the retained fragment. When the fragment remains within the peripheral venous system, surgical retrieval is often preferred. In contrast, if the fragment has migrated to central veins or the heart, endovascular retrieval techniques under fluoroscopic guidance are generally favored due to their minimally invasive nature and high success rates [13,14,15].

In the case of a pregnant patient, radiation exposure and procedural risks to the fetus must also be carefully balanced [16]. All these factors underscore the importance of prompt recognition, careful planning, and multidisciplinary coordination.

This report presents a rare case of peripheral intravenous catheter rupture in a pregnant patient and outlines the technique used for its successful retrieval. Although intravascular catheter fragments have been described in the literature, most cases involve adult or pediatric oncology patients with long-term indwelling ports or central venous lines.

To our knowledge, only three cases of peripheral intravenous cannula fracture during pregnancy have been reported in the literature. All these cases occurred in the postpartum period: two following cesarean section and one in the context of puerperal sepsis after prolonged labor. In those reports, the catheter fragments were localized using ultrasound, while plain radiography was inconclusive, and definitive management required surgical removal [17,18,19]. In a recent case series by Christian Emeka Amadi and Justina Omoikhefe Alegbeleye, 15 cases of peripheral intravenous cannula fracture were described, 13 of which occurred in pregnant women with cannulas inserted preoperatively for cesarean section. However, the fractures were identified in the postpartum period [20]. In contrast, in our case, the fragmented catheter was identified during an ongoing pregnancy at 21 weeks of gestation, which makes the management particularly challenging.

## 2. Case Presentation

A 31-year-old primipara presented at 21 weeks of gestation with a history of abundant watery vaginal discharge persisting for approximately eight days. She denied abdominal pain, uterine contractions, fever, or vaginal bleeding. Her medical, surgical, gynecological, and obstetric histories were unremarkable.

Based on clinical and ultrasound findings, the diagnosis of preterm prelabor rupture of membranes (p-PROM) at 20–21 weeks of gestation associated with severe oligohydramnios was established, and the patient was admitted for conservative management. P-PROM represents one of the most critical complications of the late second and early third trimesters, being associated with significant perinatal morbidity [21].

At admission, on 28 July 2025 at 19:00, a peripheral intravenous catheter (18 G, Gloflon^®^, (Global Medikit Limited, Selaqui, Dehradun, Uttarakhand, India)) was placed for laboratory sampling and medication administration. Cannulation was technically challenging, and the peripheral catheter was placed in the right cephalic vein in the antecubital fossa; a single reinsertion of the introducer needle occurred during the procedure. The cephalic vein was chosen for cannulation because it was more visible and accessible than the median cubital vein at the time of insertion.

Three days later, on 31 July 2025 at 22:00 o’clock, nursing staff observed leakage at the catheter site during drug administration. Inspection revealed fracture of the peripheral catheter with intravascular retention of a catheter fragment.

A chronological summary of events and interventions is presented in Table 1.

The patient remained hemodynamically and respiratory stable after the first surgical exploration, receiving daily obstetric assessments, antibiotic therapy (Cefazolin) for PROM, iron supplementation and antenatal corticosteroid therapy as gestational age advanced. Inflammatory markers were monitored regularly and remained within normal limits.

Four days later, on 05.08.2025, cardiologic evaluation including transthoracic echocardiography (preoperative) showed no evidence of intracardiac thrombus or valvular vegetations and therefore transesophageal echocardiography was not indicated.

**Table 1:** Chronological sequence of events and interventions.

Date	Events	Action
28 July 2025 19:00	Event No. 1: Peripheral intravascular catheter for blood sampling and medication	Right cephalic vein cannulation in antecubital fossa. Difficult cannulation. Single reinsertion of the introducer needle occurred during the procedure.
31 July 2025 22:00	Event No. 2: Fractured peripheral intravenous catheter observed	Vascular surgeon: ultrasonography shows intravascular fragment 1 cm above the puncture site, proximal end adherent to the venous wall (cephalic vein).
1 August 2025, 00:30–02:00	Event No. 3: First surgical exploration of the cephalic vein (brachial segment)	Exploration in OR, under local anesthesia (Lidocaine 1%, 250 mg), 2 incisions (brachial region): • First (distal) incision: longitudinal incision 3–4 cm above the peripheral catheter insertion site, with venotomy performed directly at the site of the foreign body (as identified on preoperative ultrasound performed two hours earlier). Fragment was not identified. They continued with the need for the second incision and exploration of the cephalic vein on a higher level. Ultrasound was not available at that time; therefore, the site of the second incision was determined based on palpatory examination. • Second (proximal) incision: 3 cm above the distal incision. Longitudinal incision along the lateral margin of the biceps brachii muscle in the upper arm. During venotomy, no catheter fragment was identified, and proximal migration was suspected (4 h after first ultrasound). • To prevent further migration into the central venous circulation, ligation of the cephalic vein was performed. Each longitudinal incision was approximately 1 cm long. After approximately 90 min of operative time, the intervention was discontinued, unsuccessful retrieval was declared. Additionally, as the effect of local anesthesia began to wear off, the risks and benefits of proceeding with general anesthesia in this obstetric patient were carefully weighed. Plan: repeat ultrasound, involve a more experienced surgeon.
1 August 2025 02:15	Event No. 4: Postoperative ultrasonography	Retained catheter fragment in the deltopectoral groove, with the catheter tip adherent/fixed to the venous wall (Figure 1). Owing to the sharp angulation of the cephalic arch, which impeded passage of the foreign body, and the firm adherence of the catheter fragment to the venous wall, further exploration was deferred. In addition, ligation of the cephalic vein resulted in absent flow, thereby lowering the risk of fragment migration. Plan: exploration by experienced surgeon.
5 August 2025 13:45	Event No. 5: Third ultrasonography	The intravascular catheter fragment maintained its location within the deltopectoral groove, as described on the postoperative ultrasound, associated with intraluminal thrombosis (as expected after vein ligation) of the cephalic vein surrounding the foreign body.
5 August 2025 14:00	Event No. 6: Surgical exploration by experienced vascular surgeon (deltopectoral groove)	A surgical venotomy of the cephalic vein was performed in the deltopectoral groove through a transverse incision. Following careful dissection and exposure, the cephalic vein was incised and explored intraluminal. At this level, thrombotic material was identified and direct thrombectomy was performed. Following thrombectomy, 2F Fogarty-type catheter was then advanced repeatedly through the venotomy in an antegrade direction. This maneuver allowed successful engagement and retrieval of the retained catheter fragment (Figure 2). Ligation of the cephalic vein was also performed at this level. Location: OR, under local anesthesia (Lidocaine 1% 150 mg). Duration: 35 min. For better understanding of the incision sites, please refer to Figure 3.

**Figure 1:**
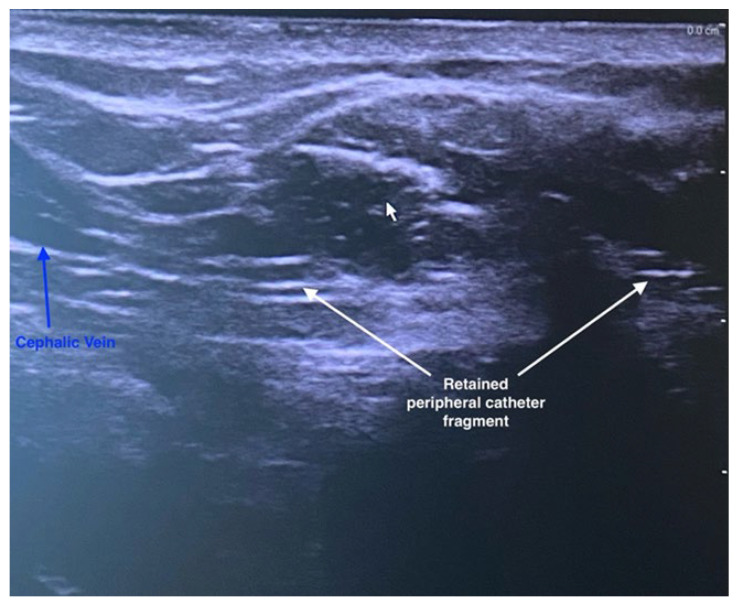
Postoperative ultrasound evaluation, after the first surgical exploration revealed the presence of a retained peripheral venous catheter fragment in the lumen of the cephalic vein, in the deltopectoral groove.

**Figure 2:**
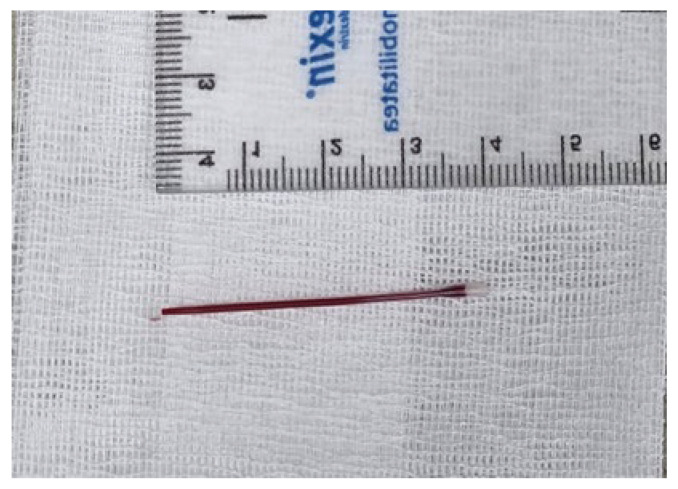
The extracted fragment shown next to a ruler indicating its length in centimeters.

**Figure 3:**
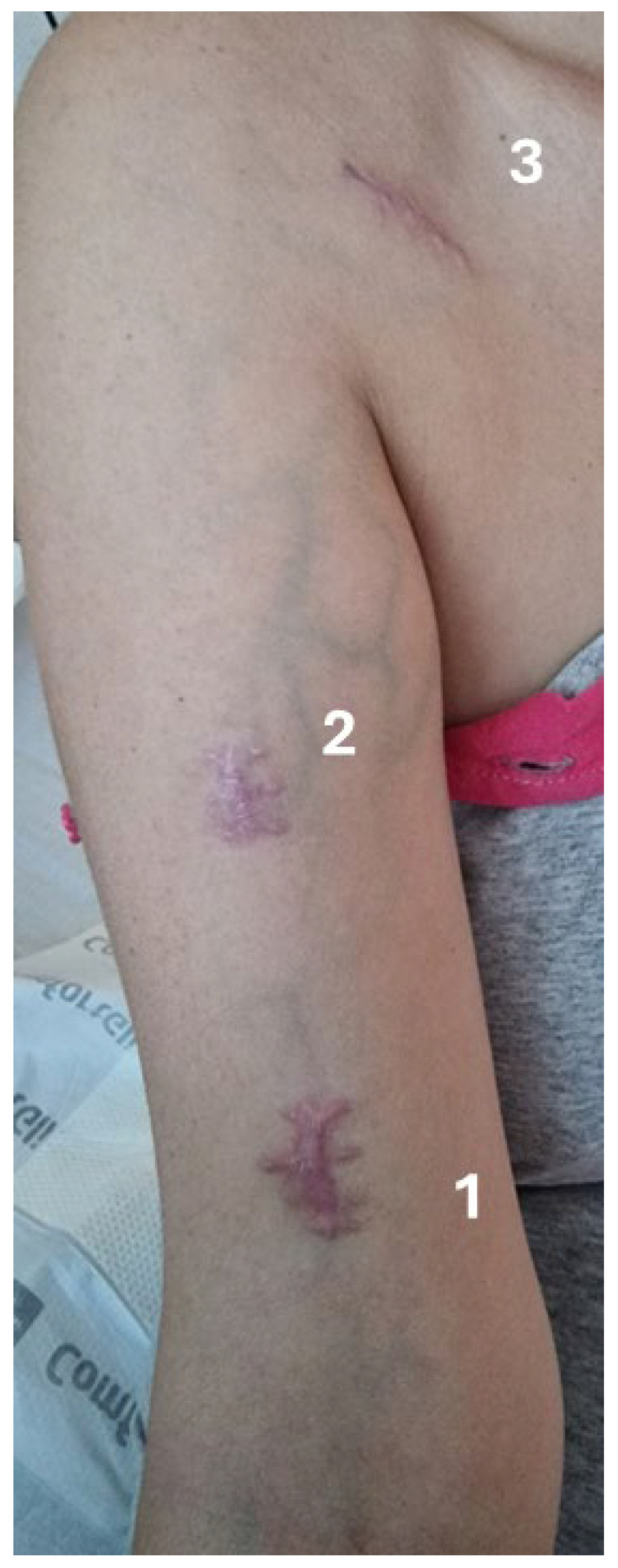
This image illustrates the incision sites. Incisions No. 1 and No. 2 were performed during the first surgical retrieval attempt, involving exploration of the cephalic vein in the brachial region. Incision No. 1 is a distal and incision No. 2 is a proximal incision. Incision No. 3 represents the second surgical attempt, consisting of exploration of the cephalic vein in the deltopectoral groove, which was successful in retrieving the intravascular catheter fragment. The image was obtained five weeks after surgery.

Following intravascular catheter fragment removal, the pregnancy progressed favorably for nine additional weeks, with no evidence of maternal infection or fetal compromise. The patient remained hospitalized for conservative management of p-PROM, receiving daily obstetric evaluations and periodic monitoring of inflammatory markers, all of which remained within normal limits.

Periodic postoperative vascular assessments of the incision site were performed. The surgical wound demonstrated normal healing progression, and sutures were removed on the sixth postoperative day.

At 29–30 weeks of gestation, spontaneous uterine contractions developed. Cervical dilation progressed rapidly and preterm vaginal delivery ensued shortly thereafter.

A preterm female neonate weighing 1400 g was delivered, with Apgar scores of 7 at 1 min and 8 at 5 min. The total duration of hospitalization was 78 days, due to the elevated risk of perinatal morbidity due to p-PROM.

## 3. Discussion

The use of peripheral cannulas has increased globally, correlating with expanding populations requiring intravenous therapy, prolonged hospitalizations, and more intensive pharmacologic regimens. Over time, complications associated with peripheral intravenous catheters have also become more apparent; meta-analyses show that mechanical, infectious, and thrombotic complications are not rare [22]. Typical local complications include phlebitis, infiltration/extravasation, occlusion, dislodgement, and leakage [23,24].

One of the most feared complications is catheter fracture with subsequent migration of the fragment. Factors predisposing to catheter fracture may be related to the catheter itself, the insertion technique, or the insertion site, while catheter fixation also plays a crucial role [25,26]. In addition, certain patient factors (e.g., small or fragile veins, obesity, edema, or anatomical constraints) may increase mechanical stress on the catheter; however, the properties of the catheter material itself also can play an important role [11,27,28].

Given the risks, several preventive measures are critical [29,30]:

Material properties: The mechanical characteristics of the catheter material (e.g., polyurethane, silicone, or other polymers) influence its flexibility, fatigue resistance, and propensity to crack under stress [31,32].

Insertion and handling protocol: Strict adherence to insertion techniques, including atraumatic puncture, minimal force, and avoiding repeated manipulation, is essential [4,33]. The use of ultrasound in vascular access has expanded significantly in recent years and is now considered an important adjunct for improving both the safety and success of venous cannulation. Conventional peripheral venous catheterization relying solely on visualization and palpation can have a first-attempt success rate as low as 51%, often requiring multiple punctures, which increases patient discomfort and the risk of mechanical complications. In contrast, ultrasound guidance has consistently demonstrated higher success rates, with prospective randomized data showing first-attempt success approaching 90% [34].

Periodic inspection and monitoring: Regular checks of catheter integrity, monitoring for signs of damage or dysfunction and routine replacement as per local protocols. Signs of catheter dysfunction include resistance during flushing, unusual pain, or local swelling [4].

Preventing overstress: Avoiding excessive bending, torque, or repeated needle recapping/manipulation techniques that can weaken the catheter wall [28].

Pregnancy is associated with several anatomical and physiological changes that may complicate peripheral venous access. Maternal blood volume increases, resulting in venous distension and altered venous wall compliance. Hormonal influences, particularly elevated progesterone levels, promote vascular smooth muscle relaxation and vasodilation [35]. Additionally, increased capillary permeability and fluid retention contribute to peripheral edema, which may obscure venous landmarks and make cannulation more challenging. In later stages of pregnancy, mechanical compression of the inferior vena cava by the gravid uterus may further alter venous return and venous pressure distribution [36]. These changes may influence both the migration of a catheter fragment and the feasibility of retrieval.

A review of the literature (Table 2) shows that peripheral intravenous catheter fracture is a rare complication, most reported in non-obstetric populations, including adults and pediatric patients. Management typically involves surgical retrieval after localization using ultrasound, radiography, or CT imaging.

In contrast, cases in obstetric patients are scarce and are almost exclusively reported in the postpartum period, most often following cesarean section. To our knowledge, no cases have been described in which catheter fracture was diagnosed and managed during an ongoing pregnancy, particularly in the second trimester.

When clinical suspicion arises, imaging and diagnostic evaluation are warranted. This typically includes plain radiography, fluoroscopy, ultrasonography, or computed tomography angiography to localize the foreign fragment, depending on the radiopaque or radiolucent properties of the catheter [43]. Once the fragment is localized, the removal strategy is planned. The choice of removal strategy is determined by fragment location, with surgical retrieval generally indicated for peripheral venous fragments and endovascular techniques preferred for centrally located fragments.

In the obstetric context, however, the choice of technique requires additional consideration, particularly in non-emergency situations. An essential consideration when managing intravascular complications in pregnant patients is adherence to the ALARA (As Low As Reasonably Achievable) principle, which guides all diagnostic imaging during pregnancy. Because the fetus is sensitive to both ionizing radiation and excessive acoustic energy, national bodies emphasize minimizing exposure while still obtaining clinically necessary information. According to the American College of Obstetricians and Gynecologists (ACOG), ultrasound and MRI are the preferred imaging modalities in pregnancy, as they are not associated with known fetal risk when used prudently, and should be employed only when they answer a relevant clinical question [44,45].

Elective procedures with minimal benefit should be deferred until at least 6–8 weeks postpartum. In emergent situations, maternal stabilization remains the priority, while in semi-urgent cases, intervention during the second or third trimester is preferable to reduce fetal radiation risk [46]. Ionizing radiation exposure during pregnancy carries risks that depend on gestational age and dose and exposure time. This effect can be deterministic, which occurs above a dose threshold (ex. teratogenicity, miscarriages). The second effect of radiation exposure is the stochastic effects, which may occur at any exposure level without a defined safe threshold (ex. carcinogenesis). Fetal exposure below 50 mGy is considered negligible, whereas doses above 100–150 mGy increase the risk of radiation-induced abnormalities, highlighting the importance of careful dose minimization during fluoroscopy-guided procedures [46,47]. Radiation risk is highest during organogenesis and early fetal development (2–15 weeks), when exposure may cause malformations or neurodevelopmental injury, while later pregnancy is less sensitive and requires higher doses to produce adverse effects [45].

The main advantage of fluoroscopy-guided retrieval is precise real-time visualization, which may increase procedural success and reduce the extent of surgical dissection. Its principal disadvantage is exposure to ionizing radiation, which, although often low, remains an important concern during pregnancy, particularly in early gestation. Most used extraction devices are the loop-snare, baskets with interwoven loops [31].

However, if endovascular retrieval is not feasible, catheter fragment location is in the peripheral venous system, surgical, vascular approaches may be required [41]. Surgical retrieval avoids radiation when performed under ultrasound guidance and may be more suitable when the fragment is superficially located or accessible through a limited venotomy. However, surgery is more invasive, may require a larger incision or longer recovery, and carries risks related to pain, bleeding, infection, and procedural stress. Therefore, in pregnant patients, the choice of retrieval method should balance maternal benefit, fetal safety, fragment location, local expertise, and the likelihood of successful minimally invasive removal.

In our case, the exact cause of the catheter fracture cannot be definitively established. However, venous cannulation was difficult, and the introducer needle was once reinserted into the catheter during the procedure. After successful insertion, the catheter was not manipulated further. Additionally, the peripheral line was placed in the right antecubital fossa, where frequent joint flexion may exert significant mechanical stress on the catheter wall, potentially contributing to catheter fatigue and fracture. Routine replacement after 48 h is not standard practice in our institution, particularly in patients with difficult venous access.

In this case, the patient had challenging cannulation, and maintaining the existing peripheral line was considered preferable to avoid repeated attempts or the need for central venous access at that stage. The catheter insertion site was regularly monitored, and no signs of local or systemic infection were observed prior to the complication.

For retrieval of the catheter fragment, a vascular surgical approach was chosen. The first exploration was unsuccessful, as the fragment could not be identified, and intraoperative ultrasound was not available at that time. During this initial procedure, the cephalic vein was ligated, resulting in cessation of blood flow and reducing the risk of migration of the fragment into the central venous system. The plan was to repeat the exploration in the presence of a more experienced surgeon, who was not available at that time.

As the cephalic vein is part of the superficial venous system, ligation resulted in cessation of blood flow, thereby reducing the risk of catheter fragment migration and predisposing to local thrombus formation, which was confirmed on follow-up ultrasonography. Owing to the presence of an extensive collateral network between the superficial and deep venous systems of the upper limb, ligation of the cephalic vein did not compromise overall venous drainage. In this context, routine anticoagulation was not prescribed, as the absence of flow limited the risk of thrombus propagation.

Transthoracic echocardiography was performed on the fourth day, mainly as a precautionary measure and to provide reassurance to the anesthesiologist and obstetrician, prior to the second intervention.

We acknowledge that there is no clear consensus regarding the optimal timing or frequency of cardiac imaging in such cases, and it remains uncertain whether more frequent (e.g., daily) evaluation would have provided additional benefit in a clinically stable patient.

During the second surgical exploration, the catheter fragment was successfully retrieved, and the procedure was completed with additional ligation of the cephalic vein at the level of the deltopectoral groove, thereby excluding the venous segment between this site and the prior mid-arm ligation.

The case resulted in a favorable outcome, with successful discharge of both the mother and the newborn.

By using real-time ultrasound rather than fluoroscopy for intravascular visualization and retrieval, the management approach in this case adhered closely to ALARA principles. It ensured effective treatment while minimizing potential fetal exposure to radiation or excessive acoustic energy. This reinforces the value of ultrasound-guided vascular procedures in pregnancy—not only for their technical feasibility and safety but also for their alignment with evidence-based imaging safety standards.

Limitations: This type of case may be subject to several limitations, including the absence of definitive ultrasound confirmation of the catheter fragment position, the lack of fluoroscopic imaging for precise localization (as local policy for pregnant patients), and the potential influence of lack operator experience on the success of first attempt to surgical retrieval. Finally, as a single case report, the findings cannot be generalized and should be interpreted within the context of an individualized clinical scenario.

## 4. Conclusions

Peripheral intravenous cannula fracture with intravascular fragment retention is a rare but potentially serious complication requiring prosmpt recognition and appropriate management. In obstetric patients, the therapeutic approach must balance effective treatment with maternal and fetal safety.

This case highlights the importance of accurate localization, the potential need for repeated or stepwise surgical intervention, and the value of experienced multidisciplinary collaboration. Ultrasound played a crucial role in guiding management and enabling successful retrieval using a Fogarty catheter after an initial unsuccessful attempt.

Cephalic vein ligation proved to be a safe adjunct to prevent further fragment migration without compromising venous drainage. The favorable maternal and neonatal outcomes emphasize that, even in complex scenarios, careful planning and tailored surgical strategies can lead to successful resolution.

## Figures and Tables

**Table 2:** The characteristics of previously reported cases of peripheral intravenous catheter fracture. In the diagnostic section, multiple imaging modalities are listed, indicating that the initial imaging technique was unsuccessful in identifying the retained intravascular fragment, and subsequent investigations were required until the fragment was successfully localized.

Author/Year of Publication	Patient Category	Catheter Rupture Site	Diagnostic	Retrieval
Lingling Pu, 2025 [8]	Neonate (1-day-old)	Left axillary vein	CT angiography	Surgical retrieval
	Infant (1-year-old)	Right temporal superficial vein	Ultrasound CT angiography	Surgical retrieval
Suresh Giragani, 2021 [37]	Adult (55 years mal)	Dorsum of right hand	Chest X-ray Multidetector CT	Endovascular retrieval
Phong Jhiew Khoo, 2018 [38]	Adult (30-year-old female)	Right dorsal metacarpal vein	Arm X-ray	Surgical removal (general surgery)
Gang Wang, 2024 [10]	Neonate (1-day-old)	Right median cubital vein	Ultrasound Echocardiography Chest X-ray	Surgical removal (sternotomy)
Aisvarya Girotra Kapoor, 2024 [39]	Adult (male, 20 s)	Right cephalic vein	CT scan-refused by the patient Ultrasound	Surgical retrieval (vascular surgery)
Peter Olalekan Adeosu, 2020 [40]	Infant (30-month-old)	Left hand dorsal vein	Ultrasound Arm X-ray CT scan	Surgical retrieval
Ayesha Masood, 2021 [41]	Adult (40 years old male)	Left cubital fossa	Arm X-ray Ultrasonography	Surgical retrieval (vascular surgery)
Chitta Ranjan Mohanty, 2018 [42]	Adult (65-year-old female)	Left external jugular vein	Ultrasound CT scan	Surgical retrieval
Nyamuryekung’e MK, 2020 [33]	Adult (76-year-old male)	Cubital fossa	Ultrasound	Surgical retrieval
Kapil Baliga, 2016 [17]	Obstetric-postpartum, post c-section	Cephalic vein	Arm X-ray Ultrasound	Surgical retrieval
Raj Ranjan Kumar, 2020 [18]	Obstetric-postpartum, post c-section	Left mid forearm	Arm X-ray Ultrasonography	Surgical retrieval
Arua, Onyinyechukwu Adaeze, 2024 [19]	Adult (23-year-old female)	Left median cubital vein	Ultrasonography	Surgical retrieval
	Obstetric-postpartum	Left cephalic vein	Ultrasonography	Surgical retrieval
Christian Emeka Amadi, 2024 [20]	13 obstetric-postpartum 1 adult (female) 1 adult (male)	Cubital fossa—10 cases Forearm—3 cases Neck—2 cases	Ultrasonography	Surgical retrieval

## Data Availability

The data used for this study can be found in the database of the Târgu Mures, County Emergency Clinical Hospital, Romania.

## Abbreviations

The following abbreviations are used in this manuscript:

p-PROM	preterm prelabor rupture of membranes
ALARA	As Low As Reasonably Achievable
